# Account of a Case, in Which a Tumor in the Spermatic Chord Was Complicated with Symptoms so Strongly Resembling Those of Incarcerated Bubonocele, as to Lead to an Operation, by Which the True Nature of the Disease Was Ascertained

**Published:** 1827-03

**Authors:** Henry Jeffreys

**Affiliations:** St. George's Hospital


### TUMOR IN THE SPERMATIC CHORD.

*Account of a Case, in which a Tumor in the Spermatic Chord was complicated with Symptoms so strongly resembling those of Incarcerated Bubonocele, as to lead to an Operation, by which the true Nature of the Disease was ascertained.* Treated at St. George's Hospital, by Henry Jeffreys, Esq.

Cases have not unfrequently occurred where another disease has been mistaken for strangulated hernia, and an operation performed for the relief of the symptoms. The most common of these has been where an enlarged absorbent gland has existed in the usual situation of rupture,—or where there has been an encysted hydrocele in the upper part of the spermatic chord,—or where the testicle has been stopped at the ring of the external oblique muscle, in its descent into the scrotum. But I do not, at this moment, recollect an instance on record exactly similar to that which forms the subject of the following history.

Philip Haplin, forty-one years of age, was admitted into St. George's Hospital, in the evening of Wednesday, December 27th, 1826, with a tumor in the left groin, accompanied by symptoms resembling those of strangulated hernia.

He said, he was a watchman, and that, on his return home on Saturday morning, December 23d, he was suddenly seized with violent pain in the belly, followed by continued nausea, having had no stool for two days before. The pain was referred principally to the navel, and the lower part of the abdomen. By the direction of a medical gentleman in the neighbourhood, who visited him, he took some pills and aperient medicine, but without any effect. On Sunday he was bled in the arm, and took more purging medicine. On Monday he was still no better, and the bleeding and purgatives were repeated. On Tuesday he observed a swelling in the left groin, and was immediately impressed with the idea that he was ruptured. In the evening of that day he had four or five watery, purging stools, but they were not followed by any material remission of the symptoms. The next morning (Wednesday) he showed the swelling in the groin to the gentleman in attendance, who confirmed his opinion of its being a rupture. The surgeon told him that he had reduced it; and, by his desire, the man walked from his residence in Chelsea to the Strand, for a truss. On his return home, he was unable to bear the pressure of the instrument: the pain and distress in the belly, together with the sickness and vomiting, became much increased; and the swelling was larger and more painful; on which account the surgeon advised that he should be immediately conveyed to the hospital. On his arrival there, the taxis was again tried, but without success. He was then put into the hot bath, and, while there, thirty-two ounces of blood were taken from his arm, when he became faint, and in that condition the house surgeon again attempted the reduction of the swelling, but with no better success than before; and I was therefore sent for.

It was between nine and ten o'clock when I got to the hospital. Upon examination, I found a tumor in the left groin, about the size of a large walnut, protruding from within the ring of the external oblique, which looked and felt, in all its apparent characters, like a strangulated bubonocele. It was firm, tense, and very painful and tender when pressed. Its anterior or projecting surface was perfectly distinct and circumscribed, and its base fixed and immoveable. The spermatic chord, from the tumor to the testicle, was free and natural. The slightest pressure excited great pain in the tumor, and increased the distress and sickness in the belly, which was swollen and distended. The man had vomited several times since his admission; appeared to be in great agony; and had a very distressed, anxious countenance. He declared positively that he had never had a swelling in that part before, and that it had become larger, and infinitely more painful, since his walk that morning to the Strand and back. I endeavoured for some time to reduce the swelling by gentle and continued pressure, but to no purpose; and therefore proceeded to the operation, as the only remaining alternative.

Upon cutting through the sheath of the chord, and dividing the fibres of the cremaster muscle, there turned out a loose elastic substance, which appeared to consist entirely of cellular membrane, and was as large as a pigeon's egg. Under the upper part of this mass, coming out from the ring, a firm white tumor, of the size of a large Spanish olive, was exposed, which was imbedded in and closely attached to the substance of the chord. It had no resemblance to a recent hernial sac, nor to the pellucid membrane of an encysted hydrocele, but was tough and thick. When cut into, about a drachm and a half of clear water escaped; and it was then perceived to consist of a cyst, the parietes of which were nearly as thick as a half-crown. Its internal surface was smooth, and formed into little sacs or pouches; and it passed up nearly half an inch within the ring, but it had no communication whatever with the abdomen. As it could not be dissected out without slitting up the ring, and at the risk of injuring some of the vessels of the chord, I laid it freely open, and, having first satisfied myself that nothing like hernia existed in any part of the inguinal canal, put some lint lightly into its cavity, and brought the upper and lower parts of the wound gently together with sutures and sticking plaster. The man was then put to bed; and, as he complained of great pain in the wound and in the belly, he had forty drops of laudanum; and was afterwards directed to take two drachms of Epsom salts, dissolved in peppermint water, every three or four hours, till stools should be obtained.

He vomited only twice after the operation: the pain in the belly then began to remit; and, in the course of the morning of the 28th, he voided four or five large purging stools, full of scybala; and could bear pressure on the abdomen without flinching. A considerable degree of swelling had already come upon the chord, which extended up within the ring and down to the testicle, and filled up all that side of the scrotum. The integuments over it were red and inflamed, and it was tender and painful when touched. His pulse was full and strong, beating 108 in the minute; and his tongue was white and furred.

Blood was taken away from his arm; and he was ordered to take a saline draught every four hours, containing half a drachm of Vinum Antimonii Tartarizati, and as much Sulphate of Magnesia.

December 29th.—He had passed plenty of purging stools, and the pain in the belly and sickness had entirely subsided. But he complained of a good deal of pain and tenderness in the chord and scrotum, the swelling of which was increased to the thickness of a man's wrist, and was tense and hard. He complained also of a troublesome cough, with an abundant expectoration of muco-puriform matter, which, he said, he had been affected with for some weeks. Tongue rather dry; pulse 108.

The sutures were cut away from the wound. Twenty leeches were directed to be applied to the swelling, and afterwards a large bread-and-water poultice, and a suspensory bandage; and the draughts to be continued.

The next day, December 30th, ten more leeches were applied. On the 31st, a healthy suppuration was established, and the lint came away from the cavity of the cyst. The swelling, pain, and tension of the chord and scrotum were much reduced; he had a clean tongue; open bowels; and his pulse was reduced to eighty-eight in a minute.

From this time nothing occurred to retard his recovery: the swelling rapidly diminished; the discharge was moderate and good; the wound filled up with healthy granulations, and on the 30th of January it was entirely healed, a very slight degree of thickening only remaining where the cyst had existed.

The close resemblance which the tumor in this case bore, in all its apparent characters, to strangulated bubonocele,—the duration and increasing severity of the symptoms,—and the total failure of the remedies already tried, left no other resource but the operation to propose: indeed, to have delayed it under such pressing circumstances would have been inexcusable. No part, however, of the relief obtained can be attributed to the operation. The complaint in the bowels was clearly obstruction and inflammation, brought on by previous constipation; which were subdued by the copious bleeding, warm bath, and other means made use of after the patient's admission into the hospital. The tumor in the groin had probably existed for some time. The man discovered it by accident, and, combining it with his other symptoms, naturally conceived that he was ruptured. The rude and ineffectual attempts to push it up into the belly, excited pain and swelling of the part, and thus rendered its resemblance to hernia more remarkable. Had the true nature of the swelling been ascertained, a better mode of treating it could not, perhaps, have been proposed than that which was adopted and proved successful,—viz. the laying it fairly open by a simple incision. The case, being one of rare occurrence, is altogether highly interesting, and its result very satisfactory.

5, *Arlington-street ; February* 1st, 1827.

